# Extreme and rapidly reversible prolongation of prothrombin time during cefoperazone/sulbactam therapy in a patient with gastrointestinal dysfunction and parenteral nutrition: a case report

**DOI:** 10.3389/fphar.2026.1854520

**Published:** 2026-06-10

**Authors:** Tian-Rui Gao, Gui-Wei Zhu, Qing-Hai Zhang, Zhen-Ping Gao

**Affiliations:** 1 School of Clinical Medicine, Shandong Second Medical University, Weifang, China; 2 Department of Critical Care Medicine, Weifang People’s Hospital, Weifang, China; 3 Department of Critical Care Medicine, Sunshine Union Hospital, Weifang, China

**Keywords:** adverse drug reaction, case report, cefoperazone/sulbactam, coagulation disorder, ICU

## Abstract

This case report describes a suspected coagulation disorder induced by cefoperazone/sulbactam administered for anti-infective therapy. The coagulopathy may be associated with the inhibition of vitamin K synthesis and utilization caused by cefoperazone/sulbactam. The patient was admitted with severe right thalamic intracerebral hemorrhage complicated by *Klebsiella pneumoniae* infection and remained on total parenteral nutrition (TPN) due to concomitant gastrointestinal dysfunction. We initiated anti-infective therapy with cefoperazone/sulbactam at a dose of 2 g administered intravenously every 12 h for seven consecutive days. On the eighth day of treatment, the patient developed severe coagulation dysfunction, with prothrombin time (PT) markedly prolonged to 262.9 s. Cefoperazone/sulbactam was immediately discontinued, and vitamin K1 along with plasma transfusion was administered. By the third day after intervention, the patient’s coagulation function significantly improved, and PT returned to normal levels. Causality assessment using the Naranjo Adverse Drug Reaction Probability Scale yielded a score of 7, indicating a probable association between the adverse event and cefoperazone/sulbactam use. This case report emphasizes that cefoperazone/sulbactam may cause extremely severe coagulation dysfunction, manifested as markedly prolonged PT in this patient. Clinicians should remain vigilant for this potential adverse reaction and closely monitor coagulation parameters during treatment.

## 1 Introduction

Cefoperazone/sulbactam (CPZ/SAM), a combination of a third-generation cephalosporin and a β-lactamase inhibitor, is widely used in clinical practice for the treatment of bacterial infections of the abdominal cavity, respiratory tract, and urinary tract (Lan et al., 2021). As a third-generation cephalosporin, cefoperazone exhibits broad-spectrum antibacterial activity. However, certain bacteria can produce β-lactamases that hydrolyze cefoperazone, thereby reducing its antimicrobial efficacy. The addition of sulbactam, a β-lactamase inhibitor, prevents the hydrolysis of cefoperazone, enhances its antibacterial activity, and broadens its antimicrobial spectrum (Huang et al., 2024). The combination demonstrates significant activity against most *Gram-positive* bacteria, *Gram-negative* bacteria, and anaerobic organisms.

Despite its clinical effectiveness, cefoperazon/sulbactam is associated with various adverse reactions, including gastrointestinal symptoms such as diarrhea, nausea, and vomiting, allergic reactions, and coagulation disorders. The reported incidence of coagulation dysfunction ranges from 19.59% to 24.39% (Shao et al., 2023; Min et al., 2025). In clinical practice, therapeutic efficacy and safety may be influenced by individual patient differences, underlying diseases, and concomitant medications. Particular caution is warranted in patients with complex comorbidities, such as hepatic or renal impairment and gastrointestinal disorders (Xu et al., 2025).

Although reports of cefoperazone/sulbactam-induced coagulopathy remain limited, the number of documented cases has been increasing in recent years. This report aims to present a representative case and to alert clinicians to this potential risk, thereby promoting early recognition and improving patient outcomes.

## 2 Case presentation

The patient was a 42-year-old male who was admitted to the emergency department due to sudden onset of left-sided limb weakness lasting 1.5 h. Cranial computed tomography (CT) revealed a right thalamic intracerebral hemorrhage with intraventricular extension. The patient had a history of hypertension for more than 1 year, with poor blood pressure control.

At admission, the patient had impaired consciousness and markedly elevated blood pressure (190/101 mmHg), and was unable to cooperate with physical examination. Initial laboratory tests revealed an elevated white blood cell (WBC) count (WBC 14.8 × 10^9^/L). After admission, he received hemostatic therapy, dehydration therapy, and neurotrophic support. However, the patient gradually developed abdominal distension, vomiting, and dyspnea. Gastrointestinal decompression yielded a large amount of coffee-ground material, suggesting upper gastrointestinal bleeding. His condition progressively deteriorated, and shock developed. After multidisciplinary discussion, the patient was transferred to the intensive care unit (ICU) for further management.

Further examinations were completed. Abdominal CT suggested possible intestinal obstruction. Coagulation tests showed elevated fibrinogen (FIB 6.73 g/L) and elevated D-dimer (2.02 mg/L). Liver and renal function tests revealed alanine aminotransferase (ALT) 85 U/L, aspartate aminotransferase (AST) 64 U/L, and total bilirubin 53.7 μmol/L, indicating liver injury and cholestasis (Table 1). The patient also had renal insufficiency, with urea 22.3 mmol/L and creatinine 209 μmol/L.

**TABLE 1:** Laboratory parameter changes of the patient.

Parameter	On the day of transfer to the ICU	On day 1 of cefoperazone/sulbactam therapy	On day 7 of cefoperazone/sulbactam therapy	Reference range
WBC(10^9^/L)	11.58	11.7	11.49	3.5–9.5
RBC(10^12^/L)	5.88	3.4	2.61	4.3–5.8
PLT(10^9^/L)	358	131	160	125–350
PT(s)	12.7	12.2	262.9	9.2–13.9
TT(s)	15.4	17.2	23.6	10.2–20.1
APTT(s)	23.2	29.6	51.1	21.2–34.8
INR	1.1	1.06	24.86	0.8–1.2
FIB(g/L)	6.73	4.05	5.28	2.0–4.0
D-dimer (μg/mL)	2.02	35.17	20.5	0.00–0.55
ALT(U/L)	85	53	33	0–50
AST(U/L)	64	44	48	0–40

The patient was treated with endotracheal intubation and mechanical ventilation, along with analgesia and sedation. Vasoactive agents were administered to maintain blood pressure and treat shock. Considering intestinal obstruction, severe abdominal distension, and gastric emptying disorder, total parenteral nutrition, enemas, and prokinetic therapy were provided. Continuous renal replacement therapy (CRRT) was initiated for renal insufficiency. Mannitol glycerol fructose and furosemide were administered to reduce intracranial pressure and alleviate cerebral edema.

After 2 weeks of treatment, the patient’s gastrointestinal function improved slightly, with restoration of bowel movements and flatus, and reduced abdominal distension. Daily monitoring of coagulation parameters showed only elevated FIB and D-dimer levels, while other indices remained within normal ranges. After stabilization of hemodynamic status, CRRT was discontinued, and the renal replacement strategy was adjusted accordingly.

One day prior to the initiation of cefoperazone/sulbactam therapy, microbiological testing identified infection with multidrug-resistant *Klebsiella pneumoniae*. Antimicrobial susceptibility testing demonstrated sensitivity to cefoperazone/sulbactam. Accordingly, intravenous cefoperazone/sulbactam (1:1) was administered at a dose of 2 g every 12 h for anti-infective treatment. On the day treatment was started, coagulation tests showed prothrombin time (PT) 12.2 s, INR 1.06, thrombin time (TT) 17.2 s, and activated partial thromboplastin time (APTT) 29.6 s, all within normal ranges. Only FIB (4.05 g/L) and D-dimer (35.17 mg/L) were elevated.

During the first 6 days of treatment with cefoperazone/sulbactam, the patient showed no clinical signs of bleeding, and no coagulation abnormalities were detected on routine monitoring.

After 7 consecutive days of treatment with cefoperazone/sulbactam, the patient developed sudden severe gastrointestinal bleeding. Emergency coagulation testing revealed PT 262.9 s, INR 24.86, TT 23.6 s, APTT 51.1 s, FIB 5.28 g/L, and D-dimer 20.5 mg/L, indicating severe coagulopathy. Serum vitamin K concentration was not directly measured; therefore, the diagnosis of vitamin K deficiency was based on functional coagulation abnormalities and the subsequent clinical response to vitamin K_1_ supplementation. Given the strong suspicion of drug-induced coagulopathy, cefoperazone/sulbactam was immediately discontinued. The patient was treated with intramuscular vitamin K_1_ at a dose of 10 mg every 12 h, along with transfusion of 300 mL fresh frozen plasma daily to rapidly replenish coagulation factors and correct the coagulopathy.

**FIGURE 1:**
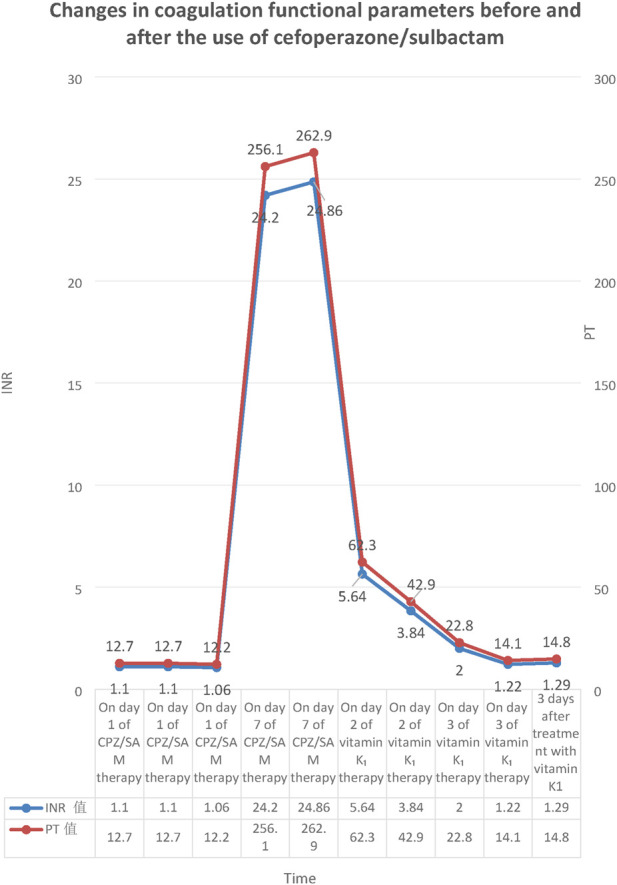
Changes in coagulation functional parameters with time after admission.

Three days after treatment with vitamin K_1_, repeat coagulation tests showed PT 14.1 s (Figure 1), INR 1.22, TT 28.8 s, APTT 29.7 s, and FIB 3.46 g/L, indicating that coagulation function had essentially returned to normal, although D-dimer remained elevated (15.69 mg/L).

## 3 Discussion

Cefoperazone/sulbactam is a broad-spectrum antibiotic widely used in the treatment of severe infections. Cefoperazone/sulbactam exhibits distinct pharmacokinetic properties that are highly relevant to the development and reversal of coagulation abnormalities. Cefoperazone has an elimination half-life of approximately 1.6–2.4 h and is predominantly excreted via the biliary tract, while only a minor proportion is eliminated renally. In contrast, sulbactam is primarily excreted through the kidneys, with an elimination half-life of approximately 1 h, which may be prolonged in patients with renal impairment. Despite its relatively short plasma half-life, cefoperazone-associated coagulopathy is not directly related to drug accumulation alone but rather to its effect on vitamin K metabolism. Cefoperazone contains an N-methylthiotetrazole (NMTT) side chain, which inhibits vitamin K epoxide reductase and interferes with the vitamin K cycle, thereby reducing γ-carboxylation of vitamin K–dependent clotting factors II, VII, IX, and X (Miao et al., 2023). This results in decreased coagulation activity and manifests clinically as prolongation of prothrombin time (PT) and elevation of the international normalized ratio (INR). In addition, cefoperazone is primarily excreted via the biliary tract. In the presence of hepatic dysfunction or cholestasis, cefoperazone may accumulate in the body, and its antimicrobial activity can disrupt normal intestinal microbiota, reducing bacterial synthesis of vitamin K (Wang et al., 2024). This further decreases endogenous vitamin K availability and exacerbates vitamin K deficiency. Therefore, normalization of coagulation parameters depends primarily on the restoration of vitamin K availability and the subsequent synthesis of functional clotting factors, rather than drug elimination alone. Although discontinuation of cefoperazone/sulbactam removes the causative agent, vitamin K supplementation plays a crucial role in accelerating recovery of coagulation function. Clinical studies and case reports have demonstrated that vitamin K administration significantly improves prolonged PT and INR in patients with cefoperazone/sulbactam-associated coagulopathy (Shao et al., 2023). These mechanisms explain why coagulation abnormalities typically occur several days after initiation of therapy and resolve following discontinuation of cefoperazone or administration of vitamin K.

In the present case, coagulation parameters were within normal ranges at baseline and remained clinically unremarkable prior to the acute bleeding event. Therefore, there was no earlier indication for discontinuation or dose adjustment before the seventh day of therapy. The patient developed sudden marked prolongation of PT (262.9 s) and elevated INR after 7 days of cefoperazone/sulbactam therapy. This clear temporal relationship strongly suggests an association between cefoperazone/sulbactam administration and coagulation dysfunction. Previous studies have shown that cefoperazone/sulbactam-associated coagulopathy typically develops within several days after initiation of therapy and is associated with significant prolongation of PT and INR (Miao et al., 2023; Wang et al., 2024; Gudivada et al., 2023; Sun et al., 2025; Chen et al., 2025). Following prompt discontinuation of the drug and administration of vitamin K1, coagulation parameters improved rapidly, further supporting the drug-related and reversible nature of this coagulopathy. Similar findings have been reported in recent case reports, in which coagulation abnormalities resolved after discontinuation of cefoperazone/sulbactam and vitamin K supplementation (Sun et al., 2025; Chen et al., 2025).

Several risk factors for vitamin K deficiency were present in this patient. Total parenteral nutrition (TPN) eliminated enteral vitamin K intake and may contribute to inadequate vitamin K supply, particularly in critically ill patients (Paulus et al., 2024), it is one of the most important contributing factors. Additionally, gastrointestinal dysfunction may impair vitamin K absorption and reduce intestinal microbial synthesis of vitamin K (Paulus et al., 2024). The patient also had renal failure requiring continuous renal replacement therapy (CRRT). CRRT is associated with significant micronutrient depletion and may increase the risk of vitamin deficiency due to extracorporeal losses and underlying critical illness (Sinatra et al., 2025; Lumlertgul et al., 2023), all of which may increase the risk of coagulation abnormalities.

Alternative causes of coagulopathy were carefully excluded. Although mild to moderate hepatic dysfunction was present, hepatic synthetic failure typically results in persistent coagulation abnormalities accompanied by reduced fibrinogen levels and delayed recovery (Driever and Lisman, 2022). In this case, fibrinogen levels were not decreased, and coagulation parameters improved rapidly following administration of vitamin K1 and fresh frozen plasma, indicating that hepatic dysfunction was unlikely to be the primary cause. Disseminated intravascular coagulation (DIC) was also considered unlikely, as DIC is characterized by consumptive coagulopathy, including progressive thrombocytopenia and decreased fibrinogen levels (Müller et al., 2021), which were not observed in this patient. Although renal failure may increase bleeding risk through platelet dysfunction, it does not directly impair clotting factor synthesis. Notably, the patient had renal failure prior to cefoperazone/sulbactam therapy without coagulation abnormalities, further supporting a drug-related mechanism. Sulbactam is primarily eliminated by the kidneys, and although accumulation may occur in renal failure, there is currently no evidence that sulbactam directly causes coagulopathy. Therefore, although sulbactam accumulation may have occurred, it is unlikely to have contributed to the marked prolongation of PT observed in this patient.

Cefoperazone/sulbactam was promptly discontinued, and the patient was treated with vitamin K1 and fresh frozen plasma. Fresh frozen plasma directly replenishes coagulation factors, enabling rapid correction of coagulation abnormalities, while vitamin K1 restores endogenous synthesis of clotting factors and supports sustained recovery of coagulation function (Dahlberg et al., 2021). Coagulation parameters returned to near normal levels within 3 days of treatment, further supporting the diagnosis of cefoperazone/sulbactam-induced vitamin K–dependent clotting factor deficiency.

We applied the Naranjo Adverse Drug Reaction Probability Scale to assess this case (Naranjo et al., 1981). According to the Naranjo scale, points were assigned as follows: previous conclusive reports of this reaction (1 point), the adverse event occurred after administration of cefoperazone/sulbactam (2 points), improvement after drug discontinuation and administration of vitamin K1 (1 point), exclusion of alternative causes that could explain the reaction (2 points) and confirmation by objective laboratory evidence including markedly prolonged PT and INR (1 point). The total score was 7, corresponding to a “probable” level of causality according to the Naranjo scale (Table 2).

**TABLE 2:** Naranjo adverse drug reaction probability scale assessment.

Question	Answer	Score
Are there previous conclusive reports on this reaction?	Yes	+1
Did the adverse event appear after the suspected drug was administered?	Yes	+2
Did the adverse reaction improve after discontinuation of the drug or administration of a specific antagonist?	Yes	+1
Did the reaction reappear upon readministration?	Not performed	0
Are there alternative causes that could solely have caused the reaction?	No	+2
Did the adverse reaction recur following the administration of a placebo?	Not performed	0
Did the drug reach toxic concentrations in the blood or body fluids?	Not performed	0
Did the severity of the reaction increase with higher doses or decrease with lower doses?	Not performed	0
Did the patient have a similar reaction to this or a similar drug in the past?	Not performed	0
Were objective findings present?	Yes	+1
Total score	​	7

In addition, we evaluated causality using the WHO–Uppsala Monitoring Centre (WHO–UMC) causality assessment system, which also classified the relationship as “probable.” Based on the overall analysis—including the temporal relationship, exclusion of alternative causes, and improvement following drug discontinuation—the coagulation dysfunction observed in this patient is most consistent with cefoperazone/sulbactam–associated vitamin K–dependent coagulation factor deficiency (Table 3).

**TABLE 3:** WHO–UMC causality assessment.

WHO–UMC criterion	Findings in this case
Reasonable temporal relationship	Present
Unlikely to be explained by disease or other drugs	Present
Clinically reasonable response to withdrawal	Present
Rechallenge information	Not available
Final assessment	Probable

In the present case, although therapeutic drug monitoring (TDM) of cefoperazone/sulbactam was not performed and plasma concentration data were therefore unavailable for pharmacokinetic exposure analysis, the close temporal relationship between cefoperazone/sulbactam administration and the onset of coagulopathy, marked objective coagulation abnormalities, rapid reversal following drug discontinuation and vitamin K_1_ administration, exclusion of major alternative etiologies, and formal causality assessment collectively support a probable causal association.

In addition to coagulation abnormalities, cefoperazone/sulbactam has also been associated with other hematologic adverse effects (Ma et al., 2026), as reported in critically ill patients. Previous studies suggest that these abnormalities may be related to severe infection, organ dysfunction, and complications associated with critical illness (Gudivada et al., 2023). In the present case, the patient exhibited a marked reduction in red blood cell count; however, this was more likely attributable to gastrointestinal bleeding and underlying critical illness rather than direct drug-induced hematologic toxicity. Meanwhile, the platelet count remained relatively stable and within the normal reference range. These findings suggest that the marked prolongation of PT and INR in the present case was more likely associated with coagulation dysfunction rather than generalized hematologic toxicity.

Previous studies have shown that cefoperazone/sulbactam-associated coagulation dysfunction typically results in moderate prolongation of PT. In a large retrospective study involving 820 patients, the mean PT increased to 31.23 ± 13.63 s, and patients with coagulation dysfunction had PT values of 35.42 ± 15.86 s, indicating that PT prolongation generally occurs within the range of approximately 20–60 s (Shao et al., 2023), with severe cases occasionally exceeding 100 s (Sun et al., 2025; Hu, 2019), but not reaching the extreme level observed in this case. Such marked prolongation of PT suggests that cefoperazone/sulbactam may cause more severe coagulation abnormalities in critically ill patients with multiple risk factors for vitamin K deficiency. This case further expands the clinical spectrum of cefoperazone/sulbactam-associated coagulopathy and highlights the importance of early coagulation monitoring in high-risk patients.

## 4 Conclusion

This case demonstrates that cefoperazone/sulbactam can induce severe coagulopathy, particularly in critically ill patients with multiple risk factors for vitamin K deficiency, including renal failure, prolonged critical illness, and total parenteral nutrition. The marked prolongation of PT observed in this patient, which exceeded previously reported levels, highlights the potential for extreme coagulation abnormalities. The temporal relationship between drug administration and coagulopathy onset, exclusion of alternative causes, and rapid correction following drug discontinuation and vitamin K supplementation support a probable causal association. Clinicians should be aware of this potentially serious adverse effect and closely monitor coagulation parameters during cefoperazone/sulbactam therapy, especially in high-risk populations. Early recognition and prompt intervention, including discontinuation of the offending agent and vitamin K replacement, are essential to prevent life-threatening bleeding complications.

## Key Clinical Message (KCM)

Cefoperazone/sulbactam therapy may be associated with significant prolongation of prothrombin time, particularly in patients with gastrointestinal dysfunction, parenteral nutrition dependency, or organ dysfunction. Careful monitoring of coagulation parameters and timely vitamin K supplementation should be considered in high-risk patients.

## Data Availability

The raw data supporting the conclusions of this article will be made available by the authors, without undue reservation.

## References

[B1] Chen J. Li X. Xiong X. (2025). Severe coagulation dysfunction and active bleeding induced by cefoperazone/sulbactam in a patient with severe renal insufficiency: a case report. Eur. J. Hosp. Pharm. 10.1136/ejhpharm-2025-004475 40250970

[B2] Dahlberg S. Schött U. Eriksson E. Ä. Tahirsylaj Y. Schurgers L. Kander T. (2021). Intravenous vitamin K1 for the correction of prolonged prothrombin times in non-bleeding critically ill patients: a prospective observational study. Nutrients 13 (8), 2580. 10.3390/nu13082580 34444740 PMC8401696

[B3] Driever E. G. Lisman T. (2022). Effects of inflammation on hemostasis in acutely ill patients with liver disease. Semin. Thromb. Hemost. 48 (5), 596–606. 10.1055/s-0042-1742438 35135033

[B4] Gudivada K. K. Krishna B. Sampath S. (2023). Cefoperazone-induced coagulopathy in critically ill patients admitted to intensive care unit. Indian J. Crit. Care Med. 27 (3), 183–189. 10.5005/jp-journals-10071-24417 36960109 PMC10028720

[B5] Hu H. R. (2019). Fatal vitamin K-Dependent coagulopathy associated with cefoperazone/sulbactam: a case report. Drug Saf. Case Rep. 6 (1), 6. 10.1007/s40800-019-0100-0 31201572 PMC6570729

[B6] Huang C. Lin L. Kuo S. (2024). Comparing the outcomes of Cefoperazone/sulbactam-based and non-cefoperazone/sulbactam-based therapeutic regimens in patients with multiresistant Acinetobacter baumannii Infections-A meta-analysis. Antibiot. (Basel) 13 (9), 907. 10.3390/antibiotics13090907 39335080 PMC11428705

[B7] Lan S. H. Chao C. M. Chang S. P. Lu L. C. Lai C. C. (2021). Clinical efficacy and safety of cefoperazone-sulbactam in treatment of intra-abdominal infections: a systematic review and meta-analysis. Surg. Infect. (Larchmt). 22 (8), 763–770. 10.1089/sur.2020.468 33625294

[B8] Lumlertgul N. Cameron L. K. Bear D. E. Ostermann M. (2023). Micronutrient losses during continuous renal replacement therapy. Nephron 147 (12), 759–765. 10.1159/000531947 37611551

[B9] Ma Q. Liang Q. Liu S. Chen J. Wang J. Zhang T. (2026). Cefoperazone/sulbactam-induced haemolytic anaemia and thrombocytosis: a case report and literature review. Br. J. Clin. Pharmacol. 92 (5), 1493–1499. 10.1002/bcp.70422 41555837

[B10] Miao W. Guo J. Cheng H. Zhao Q. (2023). Risk factors for Cefoperazone/sulbactam-induced coagulation disorder. Infect. Drug Resist 16, 6277–6284. 10.2147/IDR.S429706 37766881 PMC10520255

[B11] Min M. Zeng J. Zou M. Peng Y. (2025). Construction and validation of a nomogram prediction model for the risk of cefoperazone sodium/sulbactam sodium-related coagulation disorders. Infect. Drug Resist 18, 3859–3866. 10.2147/IDR.S534366 40766047 PMC12323872

[B12] Müller M. C. A. Dujardin R. W. G. Thachil J. van Mierlo G. Zeerleder S. S. Juffermans N. P. (2021). The relation between fibrinogen level, neutrophil activity and nucleosomes in the onset of disseminated intravascular coagulation in the critically ill. J. Intern Med. 290 (4), 922–927. 10.1111/joim.13346 34137469

[B13] Naranjo C. A. Busto U. Sellers E. M. Sandor P. Ruiz I. Roberts E. A. (1981). A method for estimating the probability of adverse drug reactions. Clin. Pharmacol. Ther. 30 (2), 239–245. 10.1038/clpt.1981.154 7249508

[B14] Paulus M. C. Drent M. Kouw I. W. K. Balvers M. G. J. Bast A. van Zanten A. R. H. (2024). Vitamin K: a potential missing link in critical illness-a scoping review. Crit. Care 28 (1), 212. 10.1186/s13054-024-05001-2 38956732 PMC11218309

[B15] Shao X. Ren Y. Xie N. Fan K. Sun H. Lu J. (2023). Effect of cefoperazone/sulbactam on blood coagulation function in infected emergency department patients and the necessity of vitamin K1 (VK1) preventive intervention: a single-center, retrospective analysis. Med. Sci. Monit. 29, e939203. 10.12659/MSM.939203 37271979 PMC10251771

[B16] Sinatra N. Maniaci A. Cuttone G. Senussi Testa T. Tutino S. Paternò D. S. (2025). Personalized parenteral nutrition in critically ill patients undergoing continuous renal replacement therapy: a comprehensive framework for clinical practice. J. Pers. Med. 15 (11), 545. 10.3390/jpm15110545 41295247 PMC12653799

[B17] Sun S. Wang Y. Min J. Wang Z. Yu L. (2025). Severe delayed coagulopathy caused by cefoperazone/sulbactam: a case report. J. Int. Med. Res. 53 (10), 3000605251375259. 10.1177/03000605251375259 41034709 PMC12665818

[B18] Wang Q. Liang P. Xu Y. Yuan B. Lan C. Yan X. (2024). Serum trough concentration threshold and risk factors of cefoperazone-induced coagulopathy in critically ill patients: a retrospective case-control study. Eur. J. Clin. Pharmacol. 80 (5), 737–746. 10.1007/s00228-024-03634-4 38353692 PMC11001783

[B19] Xu C. Zhu J. Tu K. Tang H. Zhou X. Li Q. (2025). The clinical features and risk factors of coagulopathy associated with cefoperazone/sulbactam: a nomogram prediction model. Front. Pharmacol. 15, 1505653. 10.3389/fphar.2024.1505653 39830359 PMC11742127

